# Results of a prospective dose intensity and neutropenia prophylaxis evaluation programme (DIEPP) in cancer patients at risk of febrile neutropenia due to myelosuppressive chemotherapy

**DOI:** 10.1007/s00508-015-0917-1

**Published:** 2016-01-08

**Authors:** Radosław Mądry, Lidia Popławska, Ferdinand Haslbauer, Martin Šafanda, Doru Ghizdavescu, Jana Benkovicova, Tibor Csőszi, Georgi Mihaylov, Daniela Niepel, Christine Jaeger, Iveta Frkanova, Alina Macovei, Christine Staudigl

**Affiliations:** 1 Clinic of Oncology, Poznan University of Medical Sciences, Szamarzewskiego 82/84, 60-569 Poznan, Poland; 2 The Maria Sklodowska-Curie Memorial Cancer Center, Warsaw, Poland; 3 Landeskrankenhaus Vöcklabruck, Vöcklabruck, Austria; 4 Na Homolce Hospital, Prague, Czech Republic; 5 Ploiesti Municipal Hospital, Ploiesti, Romania; 6 Oncology clinic, St. Vincent s.r.o, Prievidza, Slovak Republic; 7 Jász-Nagykun-Szolnok County Hospital, Szolnok, Hungary; 8 Specialized Hospital for Active Treatment of Haematological Diseases, Sofia, Bulgaria; 9 Amgen GmbH, Head Office for Central & Eastern Europe, Vienna, Austria; 10 Amgen Austria GmbH, Vienna, Austria; 11 Amgen Slovakia s.r.o, Piestany, Slovak Republic; 12 Amgen Romania Srl, Bucharest, Romania; 13 Medical University of Vienna, Vienna, Austria

**Keywords:** Febrile neutropenia, Neoplasms, Chemotherapy, Granulocyte colony-stimulating factor, Observational study

## Abstract

**Objective:**

To describe the incidence of febrile neutropenia (FN) and use of pegfilgrastim in cancer patients with high overall risk of FN and to investigate the relationship between granulocyte-colony stimulating factor (G-CSF) guideline adherence and chemotherapy delivery in Central and Eastern Europe (CEE) and Austria.

**Methods:**

Dose Intensity Evaluation Program and Prophylaxis (DIEPP) was a multicentre, prospective, and observational study of adult patients with breast cancer, lymphoma, lung cancer, gastric cancer, and ovarian cancer, who received chemotherapy with pegfilgrastim support and who had an overall risk of FN ≥ 20 %. Physicians assessed patient risk factors and reported their reasons for administering pegfilgrastim.

**Results:**

Patients were enrolled from 113 centres in CEE and Austria between August 2010 and July 2013, and data were analysed from 1072 patients. The most common tumour types were breast cancer (50 %) and lymphoma (24 %). FN incidence was 5 % overall. FN occurred in 3 % of patients (28/875) who received pegfilgrastim as primary prophylaxis (PP) and 13 % of patients (19/142) who received it as secondary prophylaxis (SP); 79 % of FN events in SP patients occurred in the first cycle before pegfilgrastim was administered. The three most frequently chosen reasons for using pegfilgrastim were planned chemotherapy with high FN risk, female gender, and advanced disease. Overall, 40 % of patients received > 90 % of their planned chemotherapy dose within 3 days of the planned schedule.

**Conclusion:**

FN incidence was relatively low with pegfilgrastim PP in patients with a physician-assessed overall FN risk of ≥ 20 %. The most important reasons for pegfilgrastim use were consistent with the investigators’ risk assessment and international guidelines.

## Introduction

Neutropenia remains a frequent, dose-limiting toxicity of cancer chemotherapy and carries the risk of life-threatening infections, which may compromise patient outcomes [1–3]. Specifically, febrile neutropenia (FN) is considered a medical emergency with a risk of mortality and requiring immediate hospitalisation [1–3]. Resulting dose delays and dose reductions to planned chemotherapy [4, 5] may reduce the survival of patients with potentially curable malignancies [6–10].

Primary prophylaxis (PP) with pegfilgrastim, a long-acting granulocyte-colony stimulating factor (G-CSF), improves patient outcomes by reducing the depth and duration of neutropenia, reducing infectious death during chemotherapy, and by helping planned chemotherapy dose intensity to be maintained [11–17]. The once-per-cycle dosing regimen of pegfilgrastim (6 mg, at least 24 h following chemotherapy [11]) may avoid the problem of suboptimal dosing of short-acting G-CSFs (which need to be administered as a course of daily injections) that has been found to occur in routine clinical practice [18]. Indeed, a number of studies have shown pegfilgrastim to be more efficacious than short-acting G-CSFs [19–24].

International guidelines from the American Society of Clinical Oncology (ASCO), European Organization for Research and Treatment of Cancer (EORTC), and National Comprehensive Cancer Network (NCCN) are aligned in recommending primary G-CSF prophylaxis to patients at ≥ 20 % risk of FN, either arising from the chemotherapy regimen alone, or the combination of chemotherapy regimen and individual FN risk factors [18, 25, 26]. However, implementing these guidelines in routine clinical practice has been inconsistent [5, 27–32]. There are challenges in FN risk assessment because there are chemotherapy regimens and clinical settings in which FN risk assessment is unclear in the literature [33], and work to obtain clinical tools that indicate the contribution of individual risk factors to a patient’s overall FN risk is not yet complete [34, 35]. Perhaps more concerning are reports indicating that many patients receiving curative chemotherapy do not receive G-CSF PP despite physician assessment of high FN risk [5, 27–32]. Dose reductions and dose delays are common in current practice [5, 27–32], which may be appropriate and in accordance with guidelines in the palliative setting but not in the curative setting when maintaining chemotherapy delivery is important for survival outcomes [18, 25, 26].

A study of early breast cancer (EBC) and lymphoma patients in Poland, Hungary, Czech Republic, Slovakia, and Slovenia indicated that adherence to international guidelines was particularly low in these countries of Eastern Europe [30]. G-CSF prophylaxis was rarely used, and more than half of the patients had chemotherapy reductions or delays, resulting in suboptimal relative dose intensity (RDI) [30]. These results warranted further investigation to gain a better understanding of the characteristics of patients chosen for pegfilgrastim PP and secondary prophylaxis (SP) and to expand the study to include additional tumour types and countries.

In the present study, lung, gastric, and ovarian cancer were chosen, in addition to lymphoma and EBC, as representative malignant tumours that are typically treated with multidrug combination chemotherapy with myelosuppressive potential. Poland, Czech Republic, Romania, Slovakia, Slovenia, Hungary, and Bulgaria were selected as countries representative of Central and Eastern Europe (CEE), alongside Austria, whose health system is likely to be more similar to that of Western Europe [36, 37].

The aims of the current Dose Intensity Evaluation Program and Prophylaxis (DIEPP) study were to collect information on the frequency of FN and the use of pegfilgrastim in the treatment of chemotherapy-associated myelosuppression in patients with high overall risk of FN and with different cancers and to investigate the relationship between adherence to current guidelines and the degree of chemotherapy treatment compliance in CEE and Austria.

## Patients and methods

### Study design

DIEPP was a multicentre, prospective, and observational study. No laboratory or diagnostic tests, other than those performed as part of the patient’s routine care, were required. Patients were enrolled following administration of pegfilgrastim and were observed from the beginning of the first cycle of chemotherapy through the entire chemotherapy treatment period up to a maximum of eight cycles (or until the patient died, was lost to follow-up, or withdrew informed consent, whichever was sooner). Geographically representative centres across CEE and Austria were selected for patient recruitment, and sample size was calculated to ensure that the proportion of patients with FN could be estimated with sufficient precision within most subgroups. Approval of the various ethics committees was obtained according to the local laws and regulations of participating countries.

### Patients

Adult patients with EBC, diffuse large B-cell lymphoma (DLBCL), lung, gastric, or ovarian cancer, who were planned to receive more than four cycles of chemotherapy, had received pegfilgrastim according to the summary of product characteristics (SmPC) prior to initiation of the study, and who had an investigator-assessed overall risk of FN ≥ 20 % were eligible. Patients who were scheduled to receive dose-dense chemotherapy (such as CHOP-14), continuous single-agent chemotherapy, or weekly chemotherapy were excluded.

### Outcomes

The primary outcome measure was the incidence of FN in any cycle, defined as grade III or IV neutropenia with concurrent temperature ≥ 38 °C.

Secondary outcome measures included the proportion and characteristics of patients receiving pegfilgrastim PP and SP, presence of FN risk factors, most frequently selected factors contributing to the decision to use pegfilgrastim, proportion of subjects receiving full dose on schedule, proportion of breast cancer patients achieving ≥ 85 % RDI and of lymphoma patients achieving ≥ 90 % RDI, incidence of FN in the first cycle, incidence of hospitalisations associated with FN, incidence of IV anti-infective use associated with neutropenia, and safety profile of pegfilgrastim.

### Data collection and definitions

Patients were observed and data were collected from the beginning of the first chemotherapy cycle up to a maximum of eight cycles. If patients received pegfilgrastim after cycle 1 (SP), then data from prior cycles were collected retrospectively for those cycles before receiving pegfilgrastim. The last registered values before the start of treatment with pegfilgrastim were treated as baseline data. Safety-related events were collected, including adverse drug reactions (ADR) and serious adverse drug reactions (SADR) considered by the investigator as possibly related to pegfilgrastim.

Overall FN risk was assessed by physicians at baseline based on the myelotoxic potential of the chemotherapy regimen or the combination of the chemotherapy regimen and patient-related risk factors. Patient risk factors were selected by physicians from a list of the following options: age ≥ 65 years; advanced stage disease/metastases; planned antibiotic prophylaxis; prior FN; female; haemoglobin < 12 g/dl; cardiovascular disease; kidney disease; elevated liver enzymes; high dose intensity planned (≥ 80 %); bad general condition/poor nutritional status; one or more comorbidities; body surface area (BSA) < 2 m^2^; absolute neutrophil count (ANC) < 1.5 × 10^9^/l before treatment; albumin ≤ 3.5 g/dl; lymphoma histology; Asian origin; none; and other.

Reasons for pegfilgrastim usage were selected by physicians during the observation period from a drop-down list. Up to three reasons were selected in order of importance from the following list: planned chemotherapy with high risk of FN; age (≥ 65 years); advanced disease; prior FN; female; cardiovascular, kidney, or liver disease; poor performance status; poor nutritional status; anaemia; and other.

The category of pegfilgrastim use was derived programmatically as follows:

- *PP*: pegfilgrastim initiated within days 1–7 of chemotherapy cycle 1, before neutropenia or FN had occurred, and G-CSF (either pegfilgrastim or other G-CSF) continued during each subsequent cycle.
- *SP*: as above, but with pegfilgrastim initiated in cycle 2 or later and irrespective of a neutropenic event.
- *Other*: administration of pegfilgrastim that did not meet the conditions of PP or SP.

A patient was considered to have received chemotherapy that was:

- *Full dose*: if they received ≤ 10 % dose reduction from the planned amount of any myelotoxic drug in any cycle
- *On schedule*: if there was ≤ 3 days dose delay from the planned date in any cycle
- *Full dose on schedule*: if both the above criteria were met

RDI was defined as the delivered dose intensity, expressed as a percentage of the prescribed dose intensity. The RDI of a multidrug regimen was the average of the RDIs for each of the individual drugs for each regimen. If a patient received more than one regimen, the RDI was averaged among all regimens, and sequential regimens were considered as one multicomponent regimen.

### Statistical analysis

Primary and secondary outcomes were assessed using the full analysis set (FAS), which consisted of all patients who met the eligibility criteria and started a cycle of chemotherapy. All statistical analyses were descriptive in nature. For continuous variables, descriptive statistics including the mean and standard deviation (SD) were reported. For categorical variables, the number and percentage of participants in each category were reported, along with 95 % two-sided confidence intervals (95 % CI), calculated using Wilson’s formula, where appropriate.

## Results

### Study population

Patients were enrolled from 113 centres between August 2010 and July 2013. The FAS included data from 1072 patients from 111 centres in Austria (217 patients), Czech Republic (180), Poland (475), Romania (89), Slovakia (38), Slovenia (32), Hungary (24), and Bulgaria (17).

Patient and treatment characteristics are shown in Table 1. The population was generally young with good performance status. Breast cancer was the most common tumour type, accounting for 50 % of patients, followed by lymphoma (24 %), and lung (12 %), ovarian (11 %), and gastric cancer (4 %). Breast cancer patients tended to be younger and with fewer comorbidities, whereas lymphoma and lung cancer patients tended to be older and with more health-related complications. Overall, most patients had early stage cancer; however, most gastric and lung cancer patients had stage IV disease.

**Table 1:** Patient and treatment characteristics

	Breast (*N* = 536)	Lymphoma (*N* = 258)	Gastric (*N* = 38)	Ovarian (*N* = 113)	Lung (*N* = 127)	Total (*N* = 1072)
Age, median (range)	56 (25–82)	63 (23–91)	60 (39–74)	63 (38–84)	65 (30–84)	60 (23–91)
Female (%)	99	47	34	100	38	77
Body surface area, mean, m^2^	1.76	1.85	1.77	1.75	1.83	1.79
*ECOG PS (%)*						
0–1	98	76	87	89	77	89
> 1	2	24	13	11	23	11
*Cancer stage (%)*						
I–III	96	62	16	34	32	71
IV	0	38	68	28	59	22
Unknown	4	0	16	38	9	8
Prior incidence of FN	2	4	13	8	8	4
*FN risk of chemotherapy* ^*a*^ *(%)*						
< 10	1	1	3	4	2	2
10–19	16	20	8	43	46	23
≥ 20	78	79	89	50	52	73
Not assessed	4	0	0	3	0	2
*Pegfilgrastim use (%)*						
Primary prophylaxis	83	87	76	81	66	82
Secondary prophylaxis	15	7	13	13	17	13
Other	1	6	11	5	17	5

*ECOG PS* Eastern Co-operative Oncology Group Performance Status, *FN* febrile neutropenia

^a^Investigator assessed at cycle 1

In this patient population, selected for having received pegfilgrastim, the majority (82 %) received pegfilgrastim as PP, with SP and other reasons accounting for 13 and 5 %, respectively, and 91 % overall completed pegfilgrastim treatment as planned.

The proportion of patients who received chemotherapy with FN risk ≥ 20 % in cycle 1 was approximately 90 % for gastric cancer, 80 % for breast and lymphoma, and 50 % for ovarian and lung cancer. Most of the remaining patients in each tumour type received chemotherapy with an FN risk of 10–19 %, with very few receiving low-risk chemotherapy or who were not assessed. Patient FN risk factors at baseline are shown in Table 2. The most frequently cited FN risk factors were female gender, advanced disease/metastasis, BSA < 2 m^2^, high planned dose intensity (≥ 80 %), age ≥ 65 years, and haemoglobin < 12 g/dl. Baseline risk factors were more common in patients given pegfilgrastim as PP than those given SP, particularly advanced disease, high planned dose intensity, BSA < 2 m^2^, and cardiovascular comorbidities. SP patients were more likely to have had prior FN and low baseline ANC (Table 2), but since baseline data were defined as those taken immediately before pegfilgrastim initiation, in SP patients these risk factors would have been recorded after receipt of at least one chemotherapy cycle in the current course.

**Table 2:** Patient risk factors for febrile neutropenia at baseline

Risk factor	Overall, *n* (%) *N* = 1072	PP, *n* (%) *N* = 875	SP, *n* (%) *N* = 142
Age (≥ 65 years)	235 (22)	192 (22)	32 (23)
Advanced disease/metastases	369 (34)	309 (35)	40 (28)
Planned antibiotic prophylaxis	25 (2)	22 (3)	1 (1)
Prior FN	53 (5)	28 (3)	24 (17)
Female	727 (68)	624 (71)	86 (61)
Hb < 12 g/dl	212 (20)	172 (20)	30 (21)
Cardiovascular disease	142 (13)	130 (15)	6 (4)
Kidney disease	25 (2)	24 (3)	1 (1)
Elevated liver enzymes	31 (3)	25 (3)	4 (3)
High dose intensity planned (≥ 80 %)	236 (22)	212 (24)	18 (13)
Bad general condition/poor nutritional status	143 (13)	119 (14)	14 (10)
One or more comorbidities	63 (6)	54 (6)	7 (5)
BSA < 2 m^2^	318 (30)	285 (33)	25 (18)
ANC < 1.5 × 10^9^/l before treatment	35 (3)	17 (2)	14 (10)
Albumin ≤ 3.5 g/dl	33 (3)	32 (4)	1 (1)
Lymphoma histology	149 (14)	131 (15)	13 (9)
Asian origin	2 (0.2)	2 (0.2)	0 (0.0)
Other	67 (6)	37 (4)	25 (18)
Missing	35 (3)	18 (2)	0 (0)

The last registered values before the start of treatment with pegfilgrastim were treated as baseline data

*BSA* body surface area, *ANC* absolute neutrophil count, *FN* febrile neutropenia

### Incidence of febrile neutropenia

The incidence of FN in any cycle was 5 %, affecting 51 patients (Table 3). FN incidence was 3 % (*n* = 28) in patients receiving pegfilgrastim as PP and 13 % (*n* = 19) in patients receiving pegfilgrastim as SP. Overall, FN occurred most commonly in cycle 1 (26 of 51 patients with FN, 51 %). In the SP group, a large majority of FN events occurred before pegfilgrastim was given, with 79 % of cases (15 of 19 SP patients with FN) occurring in the first cycle of chemotherapy. In total, 32 patients (28 PP and 4 SP patients, 3 %) suffered from FN while receiving pegfilgrastim prophylaxis. FN occurred in all categories of chemotherapy FN risk and was similar across cancer types, ranging from 3 % in breast cancer to 7 % in lymphoma patients.

**Table 3:** Incidence of febrile neutropenia

Category	Any cycle				First cycle			
	*N*	Incidence of febrile neutropenia			*N*	Incidence of febrile neutropenia		
		*n*	%	(95 % CI)		*n*	%	(95 % CI)
All patients	1072	51	5	(4, 6)	1072	26	2	(2, 4)
Pegfilgrastim primary prophylaxis	875	28	3	(2, 5)	875	9	1	(1, 2)
Pegfilgrastim secondary prophylaxis	142	19	13	(9, 20)	142	15	11	(7, 17)
*FN risk of chemotherapy* ^*a*^ *(%)*								
< 10	33	2	6	(2, 20)	18	0	0	(0, 18)
10–19	288	16	6	(3, 9)	249	8	3	(2, 6)
≥ 20	787	33	4	(3, 6)	779	18	2	(1, 4)
Not assessed	28	1	4	(1, 18)	26	0	0	(0, 13)
*Cancer type*								
Breast	536	18	3	(2, 5)	536	10	2	(1, 3)
Lymphoma	258	19	7	(5, 11)	258	7	3	(1, 5)
Gastric	38	2	5	(2, 17)	38	1	3	(1, 13)
Ovarian	113	4	4	(1, 9)	113	4	4	(1, 9)
Lung	127	8	6	(3, 12)	127	4	3	(1, 8)

^a^ *N* values for FN risk of chemotherapy reflect the number of assessments. For “first cycle” data, this is equivalent to the number of patients; for “any cycle” data, some patients were assessed more than once (e.g. in the case of sequential chemotherapy regimens)

### Reasons for using pegfilgrastim

**Fig. 1:**
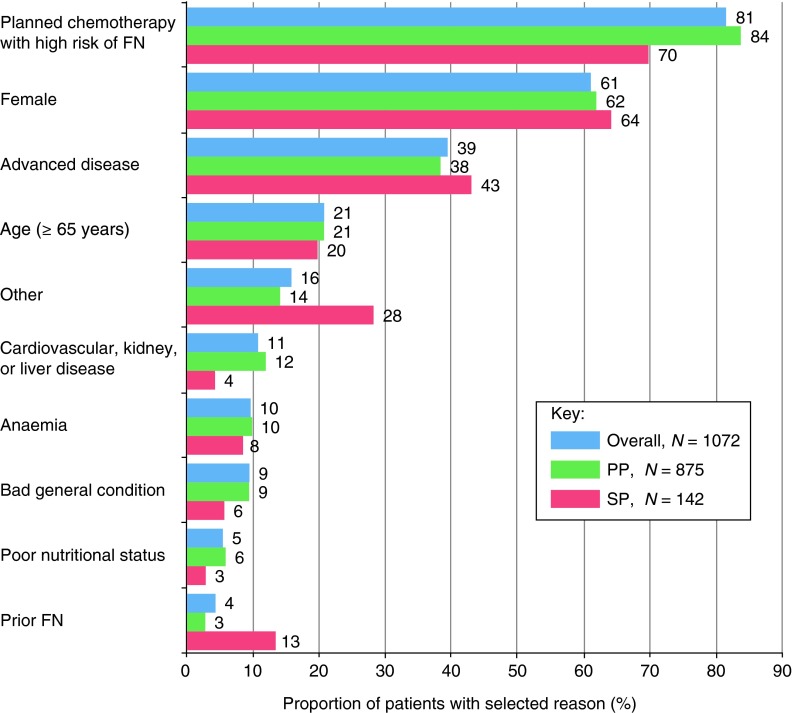
Most frequently selected factors contributing to the decision to use pegfilgrastim. Cumulative percentage frequencies are shown and represent the proportion of patients for whom each reason was selected as either first, second, or third most important

The three most frequently chosen factors contributing to the decision to use pegfilgrastim were planned chemotherapy with high risk of FN, female gender, and advanced disease, which applied to 81, 61, and 39 % of patients, respectively, overall (Fig. 1). The top three reasons were the same irrespective of whether pegfilgrastim was given as PP or SP; however, prior FN and “other” reasons were selected more often when pegfilgrastim was given as SP (13 and 28 % of patients, respectively), than when it was given as PP (3 and 14 %).

### Chemotherapy delivery

Overall, 40 % of patients received > 90 % of their planned chemotherapy dose within 3 days of the planned schedule (“full dose on schedule”; Table 4). The proportion of patients achieving full dose on schedule was highest for breast and ovarian cancer (52 and 50 %, respectively) and lowest for lymphoma (19 %). Forty-two per cent of PP patients and 32 % of SP patients received full dose chemotherapy on schedule; the proportion of patients achieving full chemotherapy delivery tended to be higher when pegfilgrastim was given as PP for all tumour types except lung cancer. The proportion of patients receiving a prespecified RDI was calculated for breast cancer and lymphoma patients: 81 % of breast cancer patients (435/536) achieved RDI ≥ 85 % and 50 % of lymphoma patients (128/258) achieved RDI ≥ 90 %.

**Table 4:** Patients achieving full dose chemotherapy on schedule

Category	Overall				Primary prophylaxis				Secondary prophylaxis			
	*N*	Full dose on schedule			*N*	Full dose on schedule			*N*	Full dose on schedule		
		*n*	%	(95 % CI)		*n*	%	(95 % CI)		*n*	%	(95 % CI)
All patients	1072	428	40	(37, 43)	875	369	42	(39, 46)	142	46	32	(25, 41)
*Cancer type*												
Breast	536	280	52	(48, 56)	445	247	56	(51, 60)	83	32	39	(29, 49)
Lymphoma	258	48	19	(14, 24)	225	45	20	(15, 26)	18	0	0	(0, 18)
Gastric	38	10	26	(15, 42)	29	8	28	(15, 46)	5	0	0	(0, 43)
Ovarian	113	57	50	(41, 60)	92	50	54	(44, 64)	15	5	33	(10, 70)
Lung	127	33	26	(19, 34)	84	19	23	(15, 33)	21	9	43	(25, 64)

The proportion of patients with chemotherapy dose reduction ≥ 10 % varied widely according to tumour type, with the highest proportion occurring in lymphoma (78 %) and lowest in breast cancer (25 %); overall, the proportion was 43 %. Compared with dose reductions, there was less variability between tumour types for the proportion of patients experiencing a dose delay by ≥ 3 days, which occurred in 41 % of patients overall and was highest for lung cancer (51 %) and lowest in breast cancer (33 %).

Technical issues were the most frequently listed reason for both dose reductions (19 % of reduced cycles) and dose delays (34 % of delayed cycles). FN was infrequently cited as a reason for dose reduction (3 cycles, 0.1 %) or delay (6 cycles, 0.5 %). Neutropenia was cited as a reason for dose reductions in 32 cycles, 2 %, although more frequently cited as a reason for dose delays (66 cycles, 6 %).

### Incidence of hospitalisations associated with neutropenia and FN

Overall, 35 patients (3 %) were hospitalised due to FN, which accounted for one third of all hospitalisations. Anti-infective treatment due to neutropenia was provided to 86 patients (8 %) overall. Anti-infective use varied with tumour type, being more frequent for lymphoma (23 %) and infrequent for the other tumour types: ovarian (2 %); breast (3 %); gastric (3 %); and lung (6 %).

### Safety

ADRs considered possibly related to pegfilgrastim were reported in 29 patients (3 %; 19 with breast cancer, 4 with lymphoma, 3 with gastric cancer, 2 with ovarian cancer, and 1 with lung cancer). The most common ADR was bone pain, which was experienced by 16 patients (1 %). One patient (0.1 %) with breast cancer experienced two SADRs considered possibly related to pegfilgrastim that occurred simultaneously (chest pain and back pain), and one lymphoma patient experienced an SADR of leucocytosis.

## Discussion

In this patient population, which was considered to be at high FN risk and provided with pegfilgrastim support, the incidence of FN was relatively low. The FN rates of incidence for the two most common tumour types in this study, breast cancer and lymphoma (3 and 7 %, respectively), were lower than those reported in observational studies in which pegfilgrastim was not an eligibility criterion and G-CSF use was lower (4–17 %, and 10–22 %, respectively) [5, 27, 30, 32, 33, 38]. However, the overall rate of FN incidence in this study (5 %) was similar to that previously reported in pegfilgrastim-treated patients in Austrian clinical practice (6 %) [33]. This magnitude of FN risk reduction appears slightly higher than expected from clinical studies, which has been shown in meta-analyses to be reduced by 70 % for pegfilgrastim PP versus placebo [19]. In general, these results show that the efficacy of pegfilgrastim observed in clinical studies is maintained in clinical practice across a broad population of tumour types and regimens, although since patient outcome is influenced by many other factors, such as the planned and received dose intensity, etc., it is difficult to draw further conclusions from a comparison of these results.

As reported in other observational studies, the PP and SP groups in this study were unbalanced with respect to risk factors and probably treatment intent [24, 27, 29, 32, 33], and comparisons between these groups should be interpreted with caution. Patients may be selected for PP over SP because they are considered to be at greater risk of experiencing FN (due to the presence of patient risk factors and/or receipt of high-risk chemotherapy) or because maintaining chemotherapy dose intensity is considered important for survival outcomes. Nevertheless, the incidence of FN was lower when pegfilgrastim was given as PP than when it was provided as SP. In fact, the data suggest that FN may have often been a trigger for providing pegfilgrastim SP, which implies that better risk assessment—and response to risk assessment—may have improved outcomes for these patients.

In observational studies of real clinical practice, such as this one, results are not only influenced by the clinical judgement of physicians but also regulations surrounding reimbursement. Out of the eight countries involved, only Hungary, representing 24 patients (2 % overall), had a national-level reimbursement restriction for pegfilgrastim, which restricted use to SP following a previous chemotherapy cycle with certain specified neutropenic events [39]. This reimbursement condition restricts Hungarian practitioners from following international guidelines [18, 25, 26]. Furthermore, restricting pegfilgrastim support to SP is particularly significant since FN has been shown to occur most frequently in the first chemotherapy cycle [40], and FN rates are lower in patients randomised to receive pegfilgrastim as PP rather than as SP or reactively following a neutropenic event [13, 14]. Although not restricted at a national reimbursement level, local protocol in Romania requires a dossier to be submitted and approved before pegfilgrastim can be prescribed (personal communication, A. Macovei). Clearly, delays caused by this administrative process have the potential to endanger patient outcomes.

The baseline characteristics of the patients in this study were as expected for a younger, healthier population, given chemotherapy treatment with curative intent that needs pegfilgrastim support. Other published observational studies concur with tumour-type patterns reported here, for example, breast cancer patients tended to be younger and healthier than other cancer types [5, 27] and gastric cancer patients tended to have more advanced stage disease [29].

Most patients (98 %) received chemotherapy with a high, or at least intermediate, risk of FN, which indicated good guideline adherence, insomuch as pegfilgrastim was not being inappropriately given to patients at low risk of FN. However, despite almost 90 % of patients with gastric cancer receiving chemotherapy with ≥ 20 % FN risk, only 76 % received pegfilgrastim as PP.

The majority of patients received pegfilgrastim as PP, which is consistent with other studies and the suggestion that SP is more commonly provided by a daily G-CSF [27, 29, 32]. Lung cancer patients had the lowest proportion of pegfilgrastim PP and highest proportion of “other” pegfilgrastim use; they also tended to be older, less healthy, and have more advanced stage disease. Perhaps in this setting it was deemed more appropriate to delay chemotherapy, and indeed, dose delays were highest in this tumour type. Once pegfilgrastim prophylaxis was initiated, most patients continued to receive it in subsequent cycles. The low proportion of discontinuation in this study (9 %) is similar to that reported from a study of US claims data, including adults diagnosed with non-Hodgkin lymphoma (NHL) or breast cancer [28] and is an important statistic, since early discontinuation of pegfilgrastim prophylaxis after the first two chemotherapy cycles has been shown to lead to unacceptably high incidence of FN [41].

The three most frequently chosen reasons for pegfilgrastim use (planned chemotherapy with high risk of FN, female gender, and advanced disease) were also the most frequently occurring FN risk factors that could be selected, indicating good alignment of the reasons for prophylaxis with actual FN risk.

Many of the most common baseline patient risk factors present in patients receiving PP were also commonly observed in the patients who received SP, for example, female gender, advanced disease/metastasis, age ≥ 65 years, and haemoglobin < 12 g/dl, raising the question of why these patients did not receive PP. Although current guidelines provide a list of individual patient risk factors that contribute to overall FN risk [18, 25, 26], a validated model that quantifies the relative contribution of specific risk factors is not currently available.

The outcome of achieving full dose on schedule, as defined in this study, was achieved by a lower proportion of breast cancer and lymphoma patients than the more commonly reported measure of chemotherapy delivery based on RDI, making it a more stringent target, but one that we consider to be clinically relevant. The proportion of breast cancer patients who achieved RDI ≥ 85 % in this study (81 %) is higher than that observed in the previous study in CEE (56 %) in which G-CSF was given to approximately half the patients [30], although similar to that reported in observational studies from other regions [4, 5, 27]. Only 50 % of lymphoma patients achieved RDI ≥ 90 % in this study. Similar to breast cancer, this again appears higher than observed in the previous CEE study (36 % of patients with RDI ≥ 85 %) but is within the range reported in other observational studies [5, 30–32]. The difference in the proportion of patients achieving full dose on schedule for breast cancer (52 %) and lymphoma (19 %) is unclear, particularly considering the importance of chemotherapy delivery in relationship to long-term outcomes is well established in both tumour types but may be related to the very aggressive therapy usually given to NHL patients. Bearing in mind the caveats of comparing PP and SP groups, a higher proportion of patients who received pegfilgrastim as PP than as SP received chemotherapy on schedule. High planned dose intensity plays a decisive role in the decision to administer PP, and it is therefore interesting that fewer patients in the SP group had planned high dose intensity, thus making dose delays more acceptable for this group. Also, important differences between countries were observed in their strategies to manage the risk of FN. The proportion of patients with chemotherapy-related FN risk ≥ 20 % receiving pegfilgrastim PP was 88 % overall (Austria 91, Czech Republic 78, Poland 93.5, and Romania 75 %). In contrast, the proportions of patients receiving full chemotherapy dose on schedule were 40 % overall (Austria 37, Czech Republic 42, Poland 49, and Romania 18 %), suggesting that reductions in chemotherapy dose intensity may have been used to decrease the FN risk, albeit with possible consequences for survival outcomes.

Despite FN occurring in a relatively low proportion of patients, the impact was nevertheless significant, causing one third of all hospitalisations in the study and affecting 35 patients. It is therefore important to ensure that high-risk patients are identified and treated according to international guidelines to prevent FN where possible.

The study was not designed to examine survival outcomes; however, appropriate G-CSF support can reduce the risk of mortality during treatment and may improve long-term survival outcomes. The administration of G-CSF is associated with improved survival, and two closely interlinked mechanisms can be identified: directly by reducing the incidence of potentially life-threatening FN [42] and indirectly by improving chemotherapy RDI and thus anti-tumour efficacy [43–45]. The risk of FN is usually highest in cycle 1 of chemotherapy. After occurrence of an FN event, chemotherapy dose is reduced and/or the administration of a new cycle delayed, both of which leads to reductions in RDI. However, it has been shown that chemotherapy drug concentration for many chemotherapeutic agents correlates directly with the rate of tumour cell eradication [6].

As with all observational studies, a lack of control for bias and the influence of confounding factors limit the ability to draw conclusions from comparisons of patient groups, particularly the PP and SP groups, within this study. Many factors influence recruitment into observational studies, and so recruitment in relation to national populations cannot be expected. In this study, recruitment across the participating countries was indeed uneven, with Hungary, Romania, and Bulgaria being particularly underrepresented in relation to the national populations. The reasons why pegfilgrastim PP was not given to patients at ≥ 20 % risk of FN, and whether local protocols may have restricted use of pegfilgrastim, were not asked for in the study. The definition of FN used was less stringent than other prospective studies and the EORTC guidelines [18] (published after study start) as it included grade III neutropenia with fever. However, events such as FN are likely to be less well documented in observational studies than clinical studies and therefore underreporting may have contributed to the low FN rates observed. The observed reductions in chemotherapy dose intensity, especially in lymphoma patients, may have been used to decrease the FN risk, albeit with possible consequences for survival outcomes. These questions would be important topics for future studies.

## Conclusions

In patients with an overall FN risk of ≥ 20 % and who had received pegfilgrastim, most patients received pegfilgrastim as PP, and incidence of FN was low (5 %). However, 60 % of the patients did not receive full chemotherapy dose on schedule, and chemotherapy delivery was lowest in lymphoma patients. The most important reasons for pegfilgrastim use were consistent with the investigators’ risk assessment and international guidelines; however, 18 % of patients did not receive pegfilgrastim PP, despite being considered to be at high risk of FN and harbouring commonly recognised FN risk factors. In those patients who received pegfilgrastim as SP, most FN occurred before pegfilgrastim support was provided; a better risk assessment and appropriate early G-CSF prophylaxis in these patients may lead to reduced incidence of FN.
